# Antibiotic resistance genes in food and gut (non-pathogenic) bacteria. Bad genes in good bugs

**DOI:** 10.3389/fmicb.2014.00754

**Published:** 2015-01-09

**Authors:** Henk Aarts, Abelardo Margolles

**Affiliations:** ^1^Department of Foodborne Infections, Centre for Zoonoses and Environmental Microbiology, National Institute for Public Health and the EnvironmentBilthoven, Netherlands; ^2^Department of Microbiology and Biochemistry of Dairy Products, Dairy Research Institute, Spanish National Research CouncilVillaviciosa, Asturias, Spain

**Keywords:** probiotics, *Lactobacillus*, bifidobacterium, commensals, antibiotic resistance genes, food, intestinal

Some ecosystems are inhabited by an extraordinarily dense population of microbes, such as fermented foods. Their microbial load can reach higher than one billion microorganisms per gram, although the bacterial diversity is normally low due to the prevalence of a few strains which rapidly adapt to a quickly changing environment. Even higher microbial numbers can be present along the human intestinal tract; however in this case the bacterial diversity is much higher comprising hundreds of different species. The large majority of bacteria inhabiting these niches is commensal and does not suppose a health risk, i.e., lactic acid bacteria in dairy fermentations or bifidobacteria in the gut of breast-fed infants. However, the close proximity of bacterial cells in such a densely populated habitats could favor the transfer of genetic material containing antibiotic resistance (AR) genes to potential pathogens, and as such could threaten human health.

Two manuscripts included in this Research Topic review current literature on AR genes present in Lactic Acid Bacteria and bifidobacteria, species which are currently used as probiotics in animal and human nutrition, or as starter cultures in fermented foods. Devirgiliis et al. (2013) report an update on AR in foodborne *Lactobacillus* and *Lactococcus* species, two genera of Lactic Acid bacteria commonly used for the fermentation of meat, milk, and vegetables. Representatives of these species are mostly non-pathogenic, but a large variety of transmissible AR genes have been identified and characterized in them. For example transfer of tetracycline resistance genes from *Lactococcus lactis* to *Enterococcus faecalis* was demonstrated. Furthermore, different *Lactobacillus* species were able to transfer erythromycin and tetracycline resistance genes to *E. faecalis*, indicating a potential risk of using Lactic Acid Bacteria starters that have not been tested for the absence of AR genes. On the other hand, the ubiquity of *tet*(W) gene in *Bifidobacterium* species is highlighted in the mini review of Gueimonde et al. (2013). *Bifidobacterium* can be the dominant bacterial population in breast-milk babies, and some species such as *B. animalis* subsp. *lactis* are extensively used in functional foods. Even though there is no experimental evidence of the transfer of AR genes from bifidobacteria to pathogenic bacteria, the transfer of *tet*(W) genes among *Bifidobacterium* species has been demonstrated.

The impact of multidrug resistant *Escherichia coli*, belonging to the group of commensal enterobacteria, isolated from animals has been tackled in this issue by Szmolka and Nagy (2013). *E. coli* has a plethora of different AR mechanisms, including efflux pumps and mobile resistance elements that allow them to adapt to environments challenged by antibiotics. In food animals, these microorganisms are a source for the spread of AR genes throughout the entire food chain, representing a public health issue. Machado et al. (2013) highlighted the role of members of the family *Enterobacteriacea* in the spread of AR. They investigated the extended-spectrum beta-lactamase (ESBL)-producing *Enterobacteriaceae* in fecal samples of healthy humans and the presence of sulfonamide resistance genes. While ESBL-producing isolates were found in 1.8% of the samples, *sul* genes were found in a very high proportion of the isolates that were sulfonamide resistant. The *sul* genes were often located in integrons, reinforcing the role of *Enterobacteriaceae* members as potential transmitters of AR genes.

Rolain (2013) and Penders et al. (2013) emphasized the potential of food and human gut as reservoirs of AR genes. Rolain compiled the sources of AR in human pathogens, i.e., food animals, aquaculture, fruits, and vegetables. Current literature support that these sources are a major factor contributing to the emergence of AR bacteria. Penders and coworkers paid special attention to the so called resistome, the whole set of AR genes found in a specific niche. They focused their review on the resistome analysis of the human gut. This has been possible thanks to the advent of next generation sequencing techniques and to the abundant metagenomic studies performed in human fecal samples. Different approaches have been used to unravel the gut resistome, such as targeted PCR-based metagenomics, functional metagenomics and sequence-based metagenomics, revealing the presence of novel genes in unculturable bacteria. However, these techniques are now starting to emerge and their application in clinic is still far from being implemented.

Djordjevic et al. (2013) offer an overview of the importance of mobile elements in spreading AR between humans, animals and soil. Commensal bacteria can facilitate transfer and amplify the reservoir of AR genes in these niches, and mobile elements seem to play a pivotal role in these processes.

In summary, the articles within this Research Topic aim to raise interest in scientists working on AR to reflect on the potential risk of the huge reservoir of AR genes that are present in various ecosystems, like those present in the human body and food. The food and gut resistomes are mainly localized in beneficial/commensal bacteria, however environmental and physiological factors could exert undesirable effects like the mobilization of these genes to be transferred into non-desirable bacteria, with the corresponding risk for human health. Thus, scientific strategies oriented to know the real risk of harboring a high AR gene load in foods and ultimately in the human gut, are urgently needed and deserve the attention of public regulatory agencies and policy makers.

## Conflict of interest statement

The authors declare that the research was conducted in the absence of any commercial or financial relationships that could be construed as a potential conflict of interest.
